# Mechanical and Electrical Properties of Rapid-Strength Reactive Powder Concrete with Assembly Unit of Sulphoaluminate Cement and Ordinary Portland Cement

**DOI:** 10.3390/ma15093371

**Published:** 2022-05-08

**Authors:** Hui Wang, Xin Cai, Chaomin Rao, Kewei Wang, Jianmin Wang

**Affiliations:** School of Civil and Environmental Engineering, Ningbo University, Ningbo 315000, China; huiwang123@aliyun.com (H.W.); caixin67@aliyun.com (X.C.); 15542496231@163.com (C.R.); wangkewei19@aliyun.com (K.W.)

**Keywords:** working performances, flexural strength, compressive strength, flexural toughness, AC electrical resistance, AC impedance spectrum

## Abstract

Fast-hardening cement can be used to quickly repair concrete constructions. Characterizing mechanical properties by electrical properties is a promising method to evaluate the mechanical performance nondestructively. However, little attention has been paid to this area. In this paper, copper-coated fine-steel-fibers-reinforced reactive powder concrete (RPC) with compound cement was manufactured. The mass ratio of sulphoaluminate and ordinary Portland cement in the compound cement was 1:1. The influence of copper-coated fine steel fibers with the volume increasing from 0 to 3.0% by the total volume of RPC on the working performances (fluidity and setting time), mechanical properties (flexural strength and toughness, drying shrinkage rate and compressive strength) and electrical parameters (AC electrical resistance and AC impedance spectroscopy curves) was investigated. The electron microscope energy spectrum experiment was applied in analyzing the macro properties of RPC. The results exhibited that the increasing volume of steel fibers led to decreasing the fluidity and retarding the setting of RPC. The electrical resistance of RPC decreased in the form of a quartic function with the volume of steel fibers. The steel fibers volume of 1.5% was the percolation threshold value. The specimens cured for 28 days showed higher electrical resistance than the specimens cured for 1 day. The flexural or compressive strength of the specimens satisfied a specific functional relationship with the volume of steel fibers and electrical resistance. The addition of steel fibers led to improving the flexural toughness and decreasing the shrinkage rate. Furthermore, 3.0% steel fibers could improve the flexural toughness by 3.9 times and decrease the shrinkage to 88.3% of the specimens without steel fibers.

## 1. Introduction

Portland cement is the main repairing cement-based material for the bridge deck of a sea crossing bridge, which has a wide range of applications, low cost and good mechanical properties and durability [1]. The bridge deck should be operated in a timely manner. However, the Portland cement usually needs to be cured for more than 3 days before it can be applied to an active surface [2]. Considering the disadvantage of the low early strength of Portland cement, fast-hardening cement came into being. Sulphoaluminate cement is a kind of rapid-hardening cement that has been applied in various major, extra-large or special projects (underwater engineering, coastal bridges, cold areas, road surface repair, etc.). However, sulphoaluminate cement shows the characteristics of late strength shrinkage, too-fast setting time, inconvenient construction, too much hydration heat, easy cracking during construction, etc. [3,4,5,6]. Due to these reasons, this cement is currently a non-mainstream variety with few manufacturers and a high cost. Therefore, it is necessary to develop the blend of Portland cement and sulphoaluminate cement.

Reactive powder concrete (RPC) is prepared based on the maximum compactness theory and is a special cement concrete material with high strength and durability [7,8]. Nowadays, there are relatively few studies on sulphoaluminate reactive powder concrete. Zhang et al. [9] studied the effects of superplasticizer, retarder, air entraining agent and mineral admixture on the structure-property and engineering application of sulphoaluminate cement RPC. Sirtoli et al. [10] investigated the shrinkage and creep of sulphoaluminate cement RPC and found that the sulphoaluminate cement RPC without any reinforcing fiber showed obvious self-drying and autogenous shrinkage. Wang et al. [11] studied the tensile strength of sulphoaluminate cement RPC and reported that the ultimate tensile strain of sulphoaluminate cement RPC reaches 4.23 times that of the normal RPC after being cured for 1 day.

Compound cement, including rapid-hardening cement and slow-setting cement, effectively combines the advantages of the two types of cement. However, little research has been conducted regarding the systematic properties of RPC with compound cement. Especially, the corresponding working and mechanical performances and the self-prediction of properties through determining the electrical parameters have not been reported.

Steel fibers, plant fibers, polypropylene fibers and carbon fibers are often used as reinforcing fiber materials of reactive powder concrete [12,13,14]. Meanwhile, for the service environment of marine concrete, ordinary steel fibers will be corroded due to long-term immersion or repeated scouring and the freeze–thaw cycles of seawater, which accelerate the deterioration and performance attenuation of concrete [15,16]. The plant fibers, polypropylene fibers and carbon fibers show excellent corrosion resistance; however, these fibers are difficult to disperse in the cement-based materials [17,18,19,20]. Copper-coated fine steel fibers show high strength and excellent toughness. Mixing copper-plated steel fibers into concrete can make the concrete possess superior mechanical properties. In addition, the steel fibers coated with copper have good corrosion resistance [21]. Using these steel fibers as the reinforcing fillers of reactive powder concrete can effectively enhance the subsequent mechanical properties and durability of concrete [22,23,24].

Copper-plated steel fiber is a kind of fiber-reinforced material that shows good electrical conductivity. Adding copper-plated steel fibers into RPC can effectively improve the corresponding conductive performance. Therefore, the electrical resistance of steel-fibers-reinforced RPC may be sensitive to its flexural or compressive strength [25], yet few researchers have paid attention to the multi-macro performances of RPC with compound cement.

In this investigation, the influence of copper-plated steel fibers on the working performances (fluidity and setting time), the electrical properties and mechanical performances of compound cement RPC was studied. This paper will provide a kind of rapid-strength cement concrete with excellent properties and special performance prediction methods in the future.

## 2. Experimental

### 2.1. Raw Materials

Sulphoaluminate cement (SAC) and ordinary Portland cement (OPC) produced by Tangshan Polar Bear Building Materials Co., Ltd., Tangshan, China and Hangzhou Haishi Cement Co., Ltd., Hangzhou, China were applied in this study. The strength grade of the two kinds of cement was 42.5 MPa. SAC shows the initial setting time of 20 min and the final of 205 min. Additionally, P·O cement shows the initial setting time and final setting time of 45 min and 10 h. In this study, quartz sand with three particle size ranges was used. Three particle size quartz sand includes coarse sand (particle sizes of 0.71~1 mm), medium sand (particle sizes of 0.35~0.59 mm) and fine sand (particle sizes of 0.15~0.297 mm). The mass ratio of coarse sand:medium sand:fine sand was 1: 1.5:0.8. Silica fume (SF) and granulated blast furnace slag powder (GGBS) were used as two kinds of mineral admixtures for improving the activity of cement-based materials. SF showed a specific surface area of 15 m^2^/g. Meanwhile, the specific surface area and density of GGBS were 436 m^2^/g and 2.9 g/cm^3^. The quartz sand, SF and GGBS were produced by Lingshou Xinhui mining processing factory, Shijiazhuang, China. Polycarboxylate-based was adopted as a high-range water-reducing agent. Li_2_SO_4_ was used as the early strength agent, and the dosage of this study was 0.15% in this test. DF-04-type polyether surfactant was used as defoamer (mixture of dry powder carrier, white to light gray powder, density of 300~450 g/L and PH of 7.0~7.5). Meanwhile, tartaric acid was used as the retarder. The purity of Li_2_SO_4_ and tartaric acid were higher than 99.9%. These three additives were provided by Yingshan New Material Technology Co., Ltd. (Shanghai, China). Copper-plated steel fibers produced by Anshan Corbett Technology Development Co., Ltd., Anshan, China were used in the experiment. Steel meshes with wire thickness of 0.8 mm and mesh size of 5.5 mm provided by Yi’an Metal Products Co., Ltd., Dongguan, China were selected for the measurement. The average length and the diameter of steel fibers were 1.5 cm and 0.2 mm. The density of steel fibers was 7.85 g/cm^3^. The particle size distribution and chemical compositions of two kinds of cement, GGBS and SF, are shown in Table 1 and Table 2.

### 2.2. Sample Preparation

The calcium formate was used to improve the early strength of ordinary Portland cement, and the mass ratio of calcium formate and ordinary Portland cement was a constant (0.7%). The mixing proportion of rapid-strength RPC is illustrated in Table 3. The contents of copper-plated steel fibers were 1, 1.5, 2, 2.5 and 3.0% by volume of RPC.

In order to prepare the reactive powder concrete, the UJZ-15 mortar produced by Beijing Zhongke Luda Test Instrument Co., Ltd., Beijing, China was used for stirring the RPC. The stirring time could be divided into the steps as follows. Firstly, all weighed dried ingredients were poured into the UJZ-15 mixer and 1 min stirring with the speed of 48 rmp/min was provided. After this mixing, the steel fibers were added and stirred for another 1 min, then a well-mixed solution of water-reducing agent and water was added to the UJZ-15 mortar stirring pot and another 6 min of stirring.

After the mixing steps were finished, the fresh RPC was used for the measurement of slump flow and the setting time. After this testing, all fresh reactive powder concrete was poured into the oil-coated molds forming the specimens with sizes of 40 × 40 × 160 mm^3^ and 50 × 50 × 50 mm^3^. The specimens of these two sizes were applied for the measurements of mechanical and electrical parameters, respectively.

### 2.3. Measurement Methods

The average value of the measuring values of the three specimens included the data for each test. Standard curing environment (20 ± 2 °C and 98% relative humidity) was provided for curing the specimens for 1 d and 28 d, respectively.

#### 2.3.1. Measurement of Basic Properties

Chinese standard GB/T2419-2005 was provided for the measurement of the slump flow of the fresh RPC [26]. The specific details could be described as the following steps.

The tabletop, tamping bar and all contact molds were wiped off by a wet cloth. After that, the fresh reactive powder concrete was poured in the testing molds in two layers. The fresh mixture was tamped 15 times with a tamping rod after each layer was poured. Then, the test mold was lifted vertically and disposed with 25 vibrations by electric jump table. Finally, the maximum diameter and its vertical diameter were tested; the corresponding average value was considered as the slump flow of fresh reactive powder concrete. The setting time of fresh reactive powder concrete was determined by Zks-100 pointer type manufactured by Hebei Xin test machine manufacturing Co., Ltd., Cangzhou, China. The measuring steps followed the Chinese standard JGJ/T 70-2009 [27].

#### 2.3.2. Measurement of Electrical Parameters

**Figure 1:**
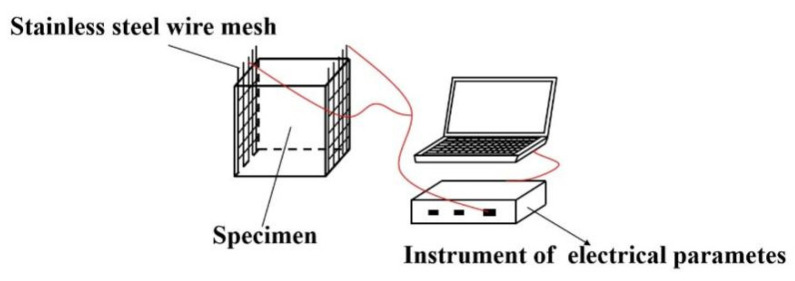
The measuring process of electrical parameters.

Figure 1 shows the measuring process of electrical parameters. The electrodes were made of 316 stainless steel mesh with square hole diameter of 4 mm. TH2810D LCR digital electric bridge (Changzhou Tonghui Co., Ltd., Changzhou, China) was used for the determination of AC electrical resistance. The testing frequencies of TH2810D were 100 Hz, 1000 Hz and 10^4^ Hz and the testing voltages were 0.1 V, 0.3 V and 1 V. PARSTAT 3000 A with the testing frequency ranging from 10^5^ Hz to 1 Hz and the magnitude of voltage of −10 mV~10 mV provided by AMETEK Trading (Shanghai) Co., Ltd., Shanghai, China was used for testing the AC impedance spectrum.

#### 2.3.3. Flexural and Compressive Strengths of Reactive Powder Concrete

**Figure 2:**
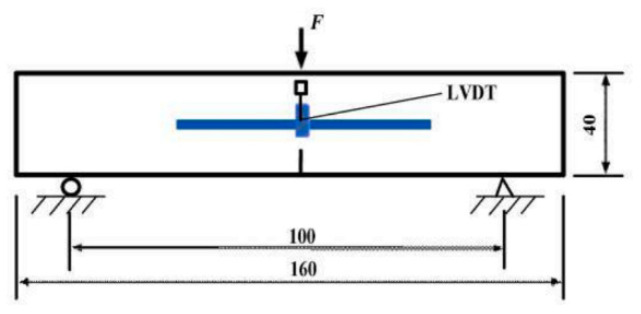
The schematic diagram of flexural loading.

The YAW 300 C microcomputer-controlled full-automatic universal testing machine manufactured by Jinan Liling testing machine Co., Ltd., Jinan, China was applied in the measurement of flexural and compressive strengths. The loading speeds for flexural and compressive strengths were 0.05 kN/s and 2.4 kN/s, respectively. The Chinese standard GB/T17671-1999 [28] was the reference standard for the test of flexural and compressive strengths. The specimens with sizes of 40 × 40 × 160 mm^3^ were used for the determination of flexural and compressive strengths. The CMT5205 microcomputer-controlled electronic universal testing machine was applied in the measurement of flexural toughness. The specimens with size of 40 × 40 × 160 mm^3^ were selected for strengths’ measurement. Linearly varying displacement transducer (LVDT) with an electronic universal testing machine produced by Shenzhen Milant Technology Co., Ltd., Shenzhen, China was used for measuring the displacement of the mid-span. The loading speed rate for the flexural toughness was 0.05 mm/min. During loading, the mid-span displacement is gradually recorded with the LVDT displacement meter. The integral of force (the value of force ranged from 0 to the peak load) and displacement curve were considered as the flexural toughness of the specimens. The detailed measuring steps could be followed in Ref. [29]. The schematic diagram of flexural loading is illustrated in Figure 2.

**Figure 3:**
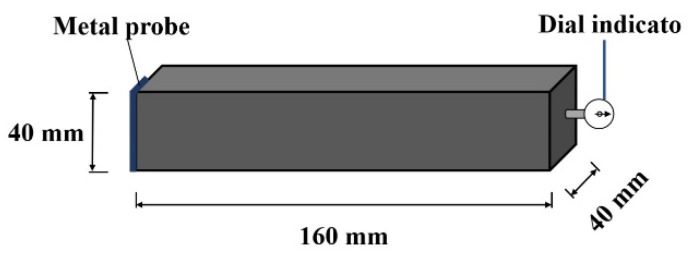
The measurement of dry shrinkage rate.

The shrinkage rod of the dial indicator, which was provided by Kaiyue Co., Ltd., Cangzhou, China, was used for measuring the dry shrinkage rate. Before the experiment, the dial indicator supported the middle of one end of the rectangular specimen. When the length of the specimen changed, the dial indicator read out the value of the length change. Through this method, the dry shrinkage rate was measured. The measurement of dry shrinkage rate is shown in Figure 3.

#### 2.3.4. Experiments of Microscopic Properties

After the curing time was reached, 5 days immersion for the samples was used for blocking hydration of cement. Some internal parts of samples with soybean size were processed in a drying oven with 105 ± 5 °C supplied by Beijing Zhongkejianyi Electronic Technology Co., Ltd., Beijing, China. After drying, the samples were taken out and sprayed with gold in vacuum environment. The samples coated with gold film were used for the measurements of scanning electron microscope (SEM) and energy dispersive spectrometer (EDS).

## 3. Results and Discussion

### 3.1. Influence of Steel Fibers on Working Performance

**Figure 4:**
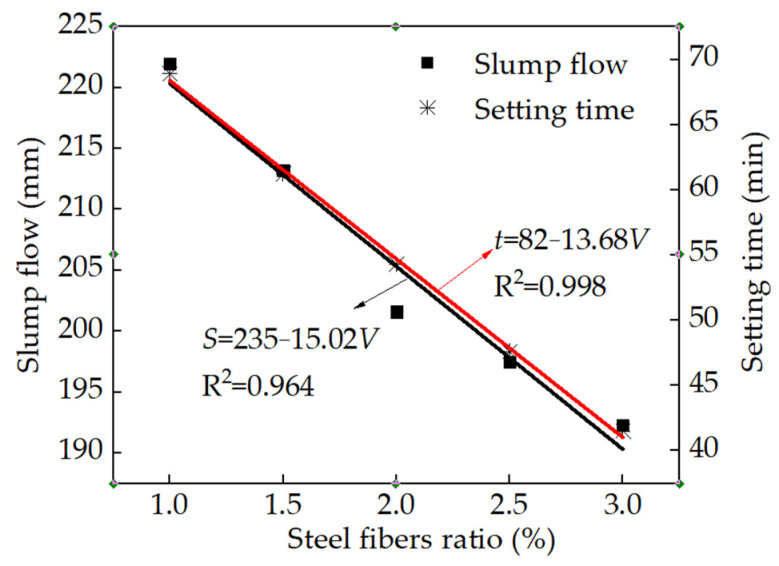
The slump flow and setting time of fresh reactive powder concrete.

Figure 4 shows the slump flow (S) and the setting time (t) of the RPC with various volume ratios of steel fibers. It can be illustrated in Figure 4 that the slump flow and setting time of the RPC decreased linearly with the increasing volume ratio (V) of steel fibers. This was attributed to the fact that the increased dosage of steel fibers agglomerated, which limited the flow of the fresh reactive powder concrete [30,31,32]. Therefore, the increasing content of steel fibers resulted in decreasing the slump flow of the fresh reactive powder concrete. The fitting degrees of the linear function were higher than 0.964, indicating the accuracy of the fitting results. Additionally, the reactive powder concrete with a high dosage of steel fibers needed more packing of cement paste, thus increasing the setting time.

### 3.2. Influence of Steel Fibers on The Electrical and Mechanical Properties

**Figure 5:**
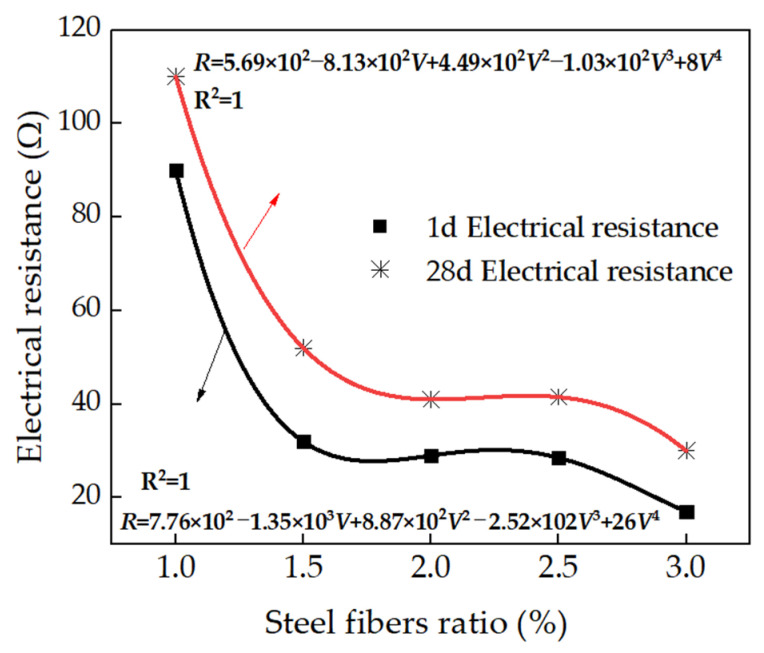
Electrical resistance of steel-fibers-reinforced reactive powder concrete.

The electrical resistance (R) of the RPC containing different contents of steel fibers is shown in Figure 5. As depicted in Figure 5, the electrical resistance dropped sharply with the volume ratio (V) of steel fibers ranging from 1 to 1.5%. This was attributed to the fact that, when the content of steel fibers is 1%, the steel fibers with low volume could not form a conductive network. Therefore, the RPC with 1% steel fibers showed low conductivity [33]. However, when the content of steel fibers reached 1.5%, the conductive networks came to connect by steel fibers and the electrical resistance decreased obviously. Meanwhile, the electrical resistance of the RPC maintained a stable level with the volume of steel fibers increasing from 1.5 to 3.0% due to the fact that, when the volume of steel fibers was around 1.5 to 3.0%, the conductive chain became integrated; hence, the electrical resistance rarely changed with the increasing volume of steel fibers. As found in Figure 5, the relationship between the electrical resistance and the volume of steel fibers corresponded to a quartic function. The fitting degree of the equations was 1.0, indicating that the fitting equation was very reasonable. Finally, Figure 5 shows that the electrical resistance of the specimens was increased by the curing age due to the improved hydration degree, which decreased the conductive ions of the inner pore solution [34,35,36,37,38].

**Figure 6:**
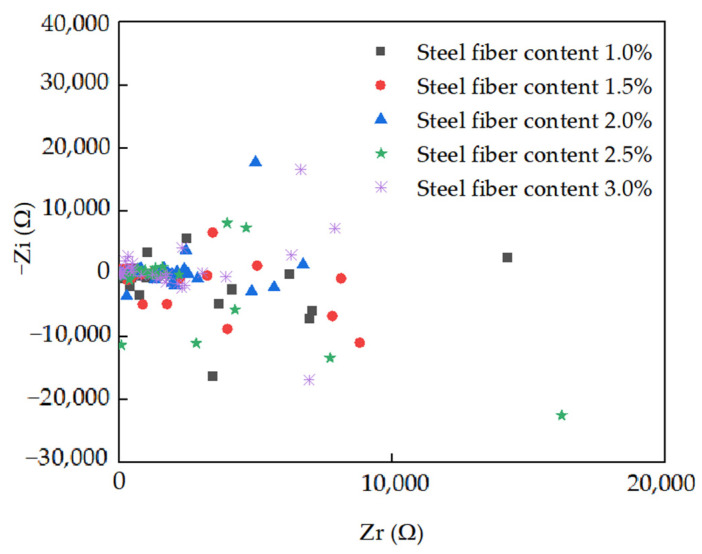
AC impedance spectrum curves.

Figure 6 shows the AC impedance spectrum curves of RPC filled with steel fibers. As depicted in Figure 6, the AC impedance spectrum curves were composed of a real part and imaginary part. The real part reflects the AC electrical resistance of the specimens with the testing frequency ranging from 10^5^ Hz to 1 Hz. As shown in Figure 6, when the content of steel fiber was lower than 2.0%, the data of the AC impedance spectrum were very discrete. This was attributed to the fact that the order of magnitude for the imaginary part of the AC impedance spectrum curves achieved 10^4^, indicating that the capacitance values of the specimens were high, thus resulting in the obvious polarization effect to the specimens [39]. Therefore, the AC impedance spectrum curves of the RPC with the steel fibers lower than 2.0% were unstable. Meanwhile, the AC impedance spectrum curves tended to be stable curves with the dosage of steel fibers ranging from 2.0 to 3.0% due to the improved electrical conductivity by the increased volume of steel fibers, which led to decreasing the polarization effect and improving the stability of the AC impedance spectrum curves [40,41,42,43,44,45].

**Figure 7:**
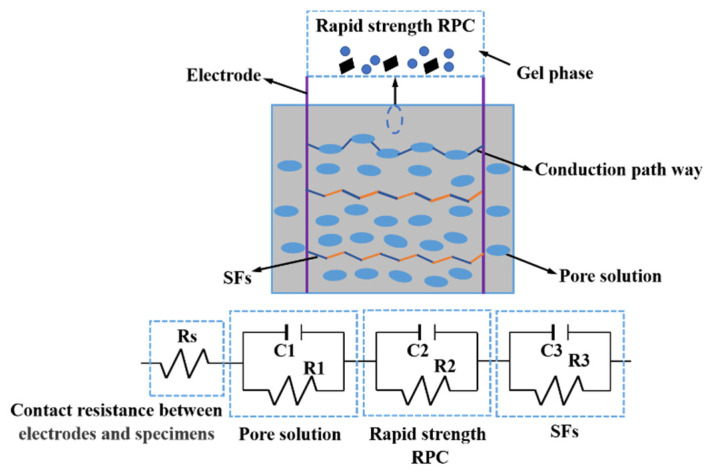
The equivalent circuit diagram.

Figure 7 shows the equivalent circuit diagram of the AC impedance spectrum curves. It can be observed from Figure 7 that the electrical properties of the steel-fibers-reinforced RPC with compound cement accorded with series conduction models of parallel electrical resistance and capacitance of pore solution, rapid-strength RPC and steel fibers, respectively, connected with the contact resistance of the electrode and specimens [46]. The chi-square value was less than or equal to 2.235 × 10^−3^, which indicates that the equivalent circuits were rational.

**Figure 8:**
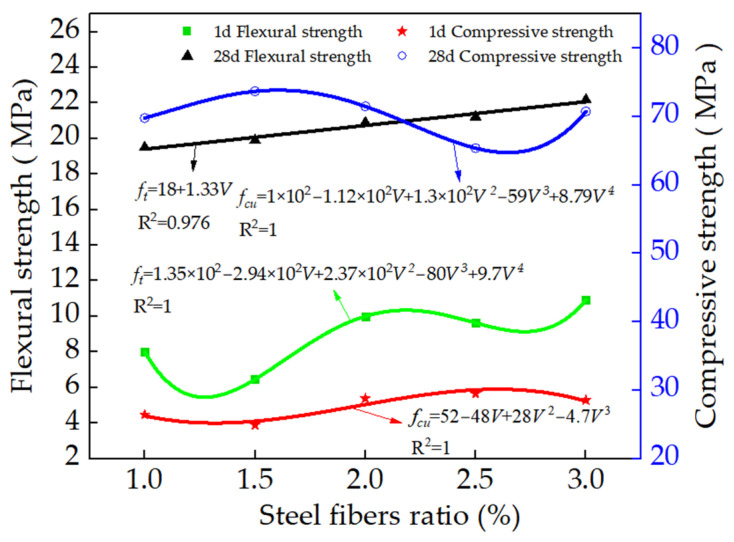
The relationship between flexural and compressive strengths and the volume of steel fibers.

Figure 8 shows the flexural strength (*f_t_*) and compressive strengths (*f*_cu_) of the steel-fibers-reinforced RPC with compound cement. It can be observed from Figure 8 that the flexural strength of the RPC cured for 28 days increased in the form of a linear function with the increasing volume of steel fibers. Meanwhile, the compressive strength cured for 28 days and flexural strength cured for 1 day varied in conformation to the quartic function. Additionally, the compressive strength of the specimens cured for 1 day can be deduced as a cubic function. The fitting degrees of all the curves were higher than or equal to 0.976; therefore, the fitting functions were consistent with the experimental results. The flexural strengths of the specimens’ days tended to increase with the increasing dosages of steel fibers due to the fact that the steel fibers could bridge the cracks in the specimen and limit the crack propagation [47,48]. Therefore, the steel fibers demonstrated a positive effect on the flexural strengths. Moreover, when the dosages of steel fibers were 1.5 and 2.5%, respectively, the compressive strengths of the specimens cured for 28 days and 1 day were the highest. This was attributed to the fact that the addition of steel fibers could limit the crack propagation, thus improving the compressive strength. However, an excessive content of steel fibers could agglomerate, leading eventually to reducing the compressive strength of the specimens [49]. The compressive strength of this kind of RPC cured for 28 days is lower than that of normal RPC due to the fact that the early hydration rate is very fast, and the generated hydration heat leads to many fine cracks in the RPC, resulting in the reduction in the strength in the later stage of RPC.

**Figure 9:**
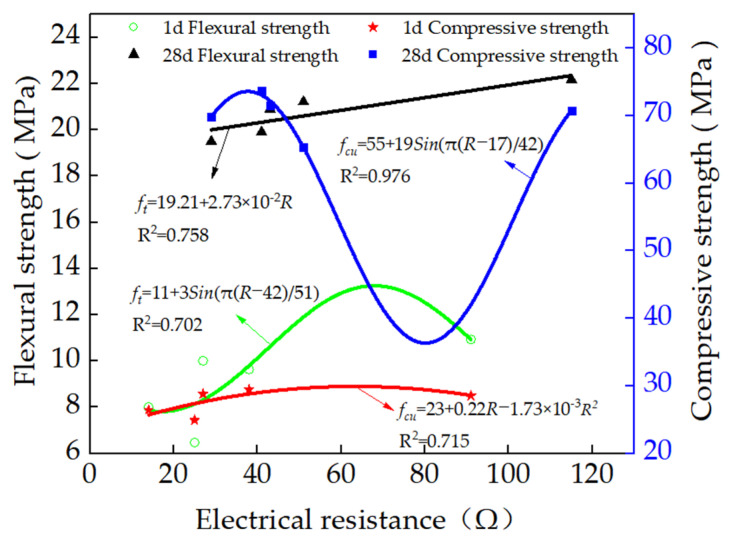
The relationship between flexural and compressive strengths and electrical resistance.

The relationships between the flexural and compressive strengths and the electrical resistance are shown in Figure 9. As depicted in Figure 9, the flexural strengths of the specimens cured for 1 day and 28 days increased in the form of a linear function. Meanwhile, the compressive strengths of the specimens varied in the quadratic function, which indicates that the electrical resistance of the specimens could be applied in sensing the flexural and compressive strengths of the specimens.

**Figure 10:**
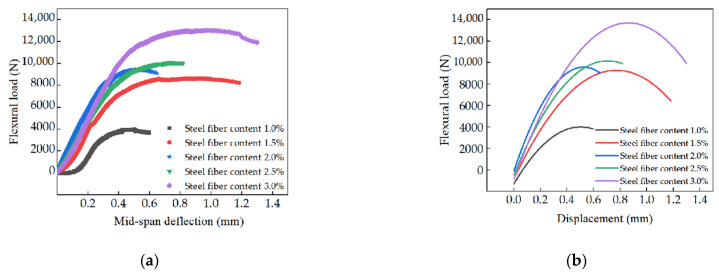
The load-mid-span deflection curves and the corresponding fitting results. (**a**) Load-mid-span deflection curves. (**b**) The fitting results load-mid-span deflection curves.

**Figure 11:**
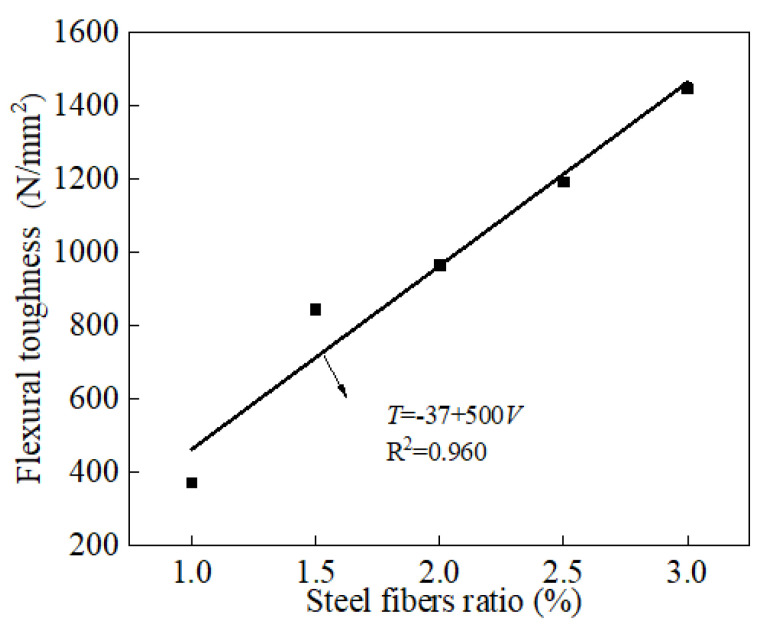
The flexural toughness of reactive powder concrete.

The load-deflection curves of the specimens under three-point bending are illustrated in Figure 10. As shown in Figure 10, the flexural load (P) increased with the deflection. The relationship between the flexural load and the deflection (γ) could be deduced as a quadratic function. Table 4 shows the fitting results of the relationship between the flexural load and the mid-span deflection. The fitting degrees of all the curves were quite high (higher than 0.952), indicating the accuracy of the fitting results. Moreover, it can be observed from Figure 10 that the maximum flexural load increased with the increasing dosages of steel fibers. Figure 11 shows the flexural toughness (T) of the specimens. It can be observed from Figure 11 that the flexural toughness increased linearly with the increasing volume of steel fibers. The fitting degree was 0.960, which ensured the accuracy of the fitting function. As can be obtained from the overall analysis, the addition of steel fibers demonstrated a positive effect on the flexural toughness of the specimens. This was attributed to the fact that the increasing volume of steel fibers could form multiple bridging actions inside the specimens, thus improving the flexural properties [50,51].

**Figure 12:**
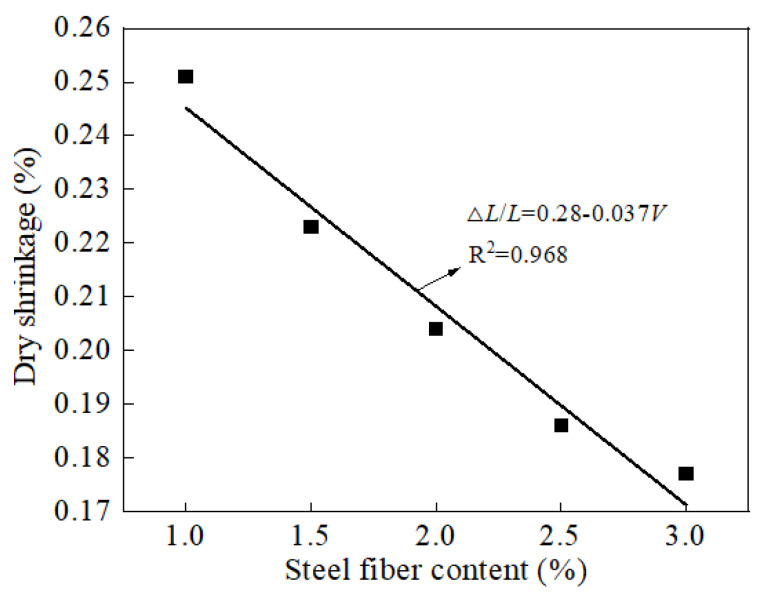
The shrinkage rate of reactive powder concrete.

Figure 12 shows the dry shrinkage rate (ΔL/L) of the specimens with the volumes ratio (V) of steel fibers ranging from 1 to 3% and cured in the standard curing environment for 28 days. As illustrated in Figure 12, the dry shrinkage rate of the specimens decreased linearly with the increasing volume of steel fibers. This was attributed to the fact that the steel fibers could limit the shrinkage of the specimens during the process of hydration [52]. Consequently, the shrinkage rate of the specimens decreased with the increasing volume of steel fibers.

### 3.3. Electron Microscope Energy Spectrum Analysis

**Figure 13:**
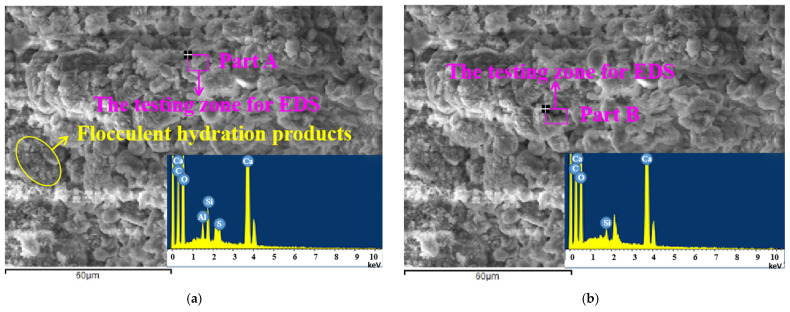

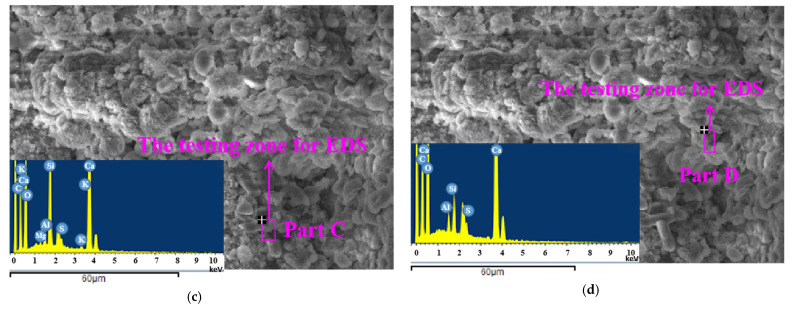
SEM-EDS of sample of four parts. (**a**) SEM-EDS of Part A. (**b**) SEM-EDS of Part B. (**c**) SEM-EDS of Part C. (**d**) SEM-EDS of Part D.

The scanning electron microscope-energy dispersive spectrometer (SEM-EDS) of the specimen without steel fibers is shown in Figure 13. The samples were cured in the standard curing environment for 28 days. As illustrated in Figure 13, several flocculent hydration products exist in the SEM photos, and the hydration products were relatively dense. Additionally, the hydration products were compact. Table 5 shows the element distribution obtained by EDS. In Table 5, parts A and D, high contents of sulfur and silicon can be observed, indicating that the part was mainly composed of hydrated calcium sulphoaluminate and hydrated calcium silicate. However, in parts B and C, the content of silicon is relatively high; therefore, these parts are mainly composed of hydrated calcium silicate and other substances.

## 4. Conclusions

The multiple properties, including the working performance, electrical parameters and the mechanical properties of reactive powder concrete with compound cement, were investigated. The research findings can be concluded as follows.

The steel fibers could decrease the slump flow and the setting time of fresh reactive powder concrete. The slump flow and the setting time decreased in the form of a linear function with the mass ratio of steel fibers.

The steel fibers could effectively improve the electrical conductivity of the reactive powder concrete. The steel fibers’ volume ratio of 1.5% was the percolation threshold value of electrical resistance. The relationship between the electrical resistance and volume ratio could be deduced as a quartic function. The specimens cured for 1 day showed higher electrical conductivity than those cured for 28 days.

The electric circuit of the reactive powder concrete accorded with series conduction models of parallel electrical resistance and capacitance of pore solution, rapid-strength RPC and steel fibers, respectively, connected with the contact resistance of the electrode and specimens.

The flexural and compressive strengths of the specimens satisfied a specific functional relationship with the volume of steel fibers and electrical resistance. The steel fibers demonstrated a positive effect on the flexural toughness and shrinkage rate. The 3.0% steel fibers could improve the flexural toughness to 3.9 times and decrease the shrinkage to 88.3% of the specimens without steel fibers.

## Figures and Tables

**Table 1:** Particle passing percentage of the cementitious materials/%.

Particle Size /μm Types	0.3	0.6	1	4	8	64	360	600
OPC	0	0.33	2.66	15.01	28.77	93.59	100	100
SAC	0	0.35	1.92	16.35	30.12	95.15	100	100
GGBS	0.025	0.1	3.51	19.63	35.01	97.9	100	100
SF	31.2	58.3	82.3	100	100	100	100	100

**Table 2:** Chemical composition of cementitious materials/%.

Types	Chemical Composition/%								
	SiO_2_	Al_2_O_3_	Fe_2_O_3_	MgO	CaO	SO_3_	T_i2_O	MnO	H_2_O
OPC	20.86	5.47	3.94	1.73	62.23	2.66	/	0	0
SAC	13.95	22.46	2.67	2.92	39.39	14.34	1.66	0	0
GGBS	34.06	14.74	0.23	9.73	35.93	0.23	3.51	0	0
SF	90	0.2	0.6	0.8	0.4	0	7.4	0	0

**Table 3:** The mixing proportions of RPC per one cubic meter (kg).

Number	Water	P·O	SAC	SF	GGBS	Quartz Sand	Water- Reducer	Li_2_SO_4_	Calcium Formate	Tartaric Acid	Defoamer	Steel Fibers
1	244.4	370.5	370.5	370.5	111.1	978.0	20.3	0.6	2.6	1.9	0.6	78.5
2	244.4	370.5	370.5	370.5	111.1	978.0	24.4	0.6	2.6	1.9	0.6	117.75
3	244.4	370.5	370.5	370.5	111.1	978.0	12.2	0.6	2.6	1.9	0.6	157
4	244.4	370.5	370.5	370.5	111.1	978.0	16.3	0.6	2.6	1.9	0.6	196.25
5	244.4	370.5	370.5	370.5	111.1	978.0	20.3	0.6	2.6	1.9	0.6	235.5

**Table 4:** The fitting results of the relationship between the flexural load and the mid-span deflection.

Equation	Steel Fibers Content/%	*a*	*b*	*c*	*R^2^*
$P=a\gamma^{2}+b\gamma +c$	1.0	−2.1 × 10^4^	2.1 × 10^4^	−1.3 × 10^3^	0.952
	1.5	−1.7 × 10^4^	2.6 × 10^4^	−8.4 × 10^2^	0.985
	2.0	−3.5 × 10^4^	3.7 × 10^4^	−1.1 × 10^2^	0.998
	2.5	−2.1 × 10^4^	3.0 × 10^4^	4.6 × 10^2^	0.998
	3.0	−2.0 × 10^4^	3.4 × 10^4^	8.8 × 10^2^	0.992

**Table 5:** The element distribution obtained by EDS (%).

Part	C	O	Mg	Al	Si	S	K	Ca
A	14.87	54.80	-	0.87	1.84	0.52	-	27.10
B	16.97	59.40	-	-	0.43	-	-	23.21
C	17.65	53.23	0.31	0.55	7.88	0.27	0.30	19.82
D	17.11	60.38	-	0.70	1.51	0.25	-	20.04

## Data Availability

The data used to support the findings of this study are available from the corresponding author upon request.
